# Disentangled Dynamic Deviation Transformer Networks for Multivariate Time Series Anomaly Detection

**DOI:** 10.3390/s23031104

**Published:** 2023-01-18

**Authors:** Chunzhi Wang, Shaowen Xing, Rong Gao, Lingyu Yan, Naixue Xiong, Ruoxi Wang

**Affiliations:** 1 School of Computer Science, Hubei University of Technology, Wuhan 430068, China; 2 Department of Computer Science and Mathematics, Sul Ross State University, Alpine, TX 79830, USA; 3 Wuhan Fiberhome Technical Services Co., Ltd., Wuhan 430205, China

**Keywords:** time series, anomaly detection, graph neural networks, transformer

## Abstract

Graph neural networks have been widely used by multivariate time series-based anomaly detection algorithms to model the dependencies of system sensors. Previous studies have focused on learning the fixed dependency patterns between sensors. However, they ignore that the inter-sensor and temporal dependencies of time series are highly nonlinear and dynamic, leading to inevitable false alarms. In this paper, we propose a novel disentangled dynamic deviation transformer network ($D^{3}TN$) for anomaly detection of multivariate time series, which jointly exploits multiscale dynamic inter-sensor dependencies and long-term temporal dependencies to improve the accuracy of multivariate time series prediction. Specifically, to disentangle the multiscale graph convolution, we design a novel disentangled multiscale aggregation scheme to better represent the hidden dependencies between sensors to learn fixed inter-sensor dependencies based on static topology. To capture dynamic inter-sensor dependencies determined by real-time monitoring situations and unexpected anomalies, we introduce a self-attention mechanism to model dynamic directed interactions in various potential subspaces influenced by various factors. In addition, complex temporal correlations across multiple time steps are simulated by processing the time series in parallel. Experiments on three real datasets show that the proposed $D^{3}TN$ significantly outperforms the state-of-the-art methods.

## 1. Introduction

Anomaly detection has been a persistent and active research direction in machine learning and has received extensive attention in various fields such as computer vision, data mining, and natural language processing (NLP). With the advent of the IoT era, more and more sensors are being placed in our surrounding environment [1], and the result of sensor acquisition is time series data [2,3]. In practical applications, it is important to be able to use this timing data to efficiently and accurately identify outliers, which helps to continuously monitor the sensor system and provide timely alerts to potential events.

A multivariate time series is composed of multiple univariate time series from the same entity, where each univariate time series represents the measured value of one sensor in the system. Traditional detection methods are designed to achieve anomaly detection by domain experts establishing corresponding thresholds based on the characteristics of univariate indicators. However, with the dramatic increase in system size and data complexity, this heavy workload and non-scalable approach have fallen off the radar. Many anomaly detection algorithms (e.g., one-class SVM [4], k-nearest neighbors [5], K-means [6]) have been proposed in recent years to overcome the drawbacks of traditional methods for anomaly detection on a single variable. Nevertheless, since indicators interact, changes in one indicator will cause fluctuations in other indicators in a complex system. Consequently, a single indicator does not represent the system’s overall state, such that these univariate detection algorithms perform poorly on multivariate time series anomaly detection tasks.

Multivariate time series anomaly detection algorithms based on deep learning have significantly improved. Hundman et al. [7] employed an LSTM-based model to capture the time dependence of multivariate time series and used the prediction bias as the anomaly score, which led to excellent results. Li et al. [8] used long and short-term memory recurrent neural networks as generators and discriminators to capture the temporal correlation of time series distributions in a GAN framework, simultaneously considering the whole collection of variables to capture the interdependencies between sensors. Although these methods have made some progress, they fail to account for the inherent relationships between sensors [9,10,11].

Recently, graph neural networks have been successful in modeling graph data, and many problems, in reality, can be abstracted to graph-structured non-Euclidean data (e.g., multimedia networks, chemical structures, traffic networks, knowledge mapping data). Multivariate time series can also be represented on a graph, where each sensor is considered a node in the graph, interconnected by hidden dependencies. Graph neural networks are better at learning patterns of inter-sensor relationships. Wu et al. [12] automatically extracted the one-way relationships between sensors by using a graph learning module and then captured the spatial-temporal dependencies in the time series using a temporal convolution module and a graph convolution module, respectively. Deng et al. [13] used a combination of structured learning and graph attention networks to automatically learn the relationship graph between sensors to capture sensor correlations and apply attention weights to account for the identified anomalies. Zhao et al. [14] separately modeled inter-sensor correlations and temporal dependencies, which captured causal links between multiple sensors as well as temporal dimensional dependencies, allowing for further anomaly detection improvements.

The aforementioned research has made some progress; however, due to the following issues, detecting anomalies based on multivariate time series remains incredibly challenging.

1. Graph convolution-based models cannot accurately describe the changes in dynamics in multivariate time series when modeling inter-sensor correlations. Spatial dependencies are highly dynamic due to unknown topologies, changing realities, and multiple factors. For each sensor, its correlated sensor varies with time step. The studies [12,13] model the dependencies between sensors by a learnable embedding of each node in the graph. Although the performance of these models has improved compared to previous deep learning models, it is still far from satisfactory. The reason is that the dependencies between sensors remain fixed after training, so it is not enough to consider only the fixed correlations of the dependencies in the graph structure during graph structure-based modeling. Moreover, for robust time series prediction, the ideal algorithm should go beyond local connectivity and extract multiscale structural features and long-range dependencies since structurally separated sensors in the learned sensor relationship graph can also have hidden correlations. Therefore, it is necessary to efficiently capture these dynamic spatial dependencies and hidden correlations to improve time series anomaly detection.
2. Long-term time dependence has often been overlooked in previous work. Long-term time dependence refers to the fact that the current state of a system may be influenced by the state of the system long ago. Gated recurrent neural networks are the most effective sequential models for practical applications, including gated recurrent units and long and short-term memory (LSTM). The papers [7,15] learn time dependence based on LSTM to capture anomalous patterns in multivariate time series. However, since the LSTM cannot adequately encode long sequences as intermediate vectors, it cannot capture temporal correlations that do not match its structure. At the same time, these models lead to time-consuming computational processes and limited scalability due to the sequential propagation characteristics. Therefore, long-term time dependence remains highly challenging in multivariate time series.

To alleviate the above challenges, we propose an unsupervised disentangled dynamic deviation transformer network ($D^{3}TN$). Specifically, this model is mainly made up of two components: a prediction module and a dynamic deviation module. The prediction module mainly consists of a feature block and a temporal block. The dependencies between sensors are complex and variable. The inter-sensor dependencies over time can be decomposed into static components determined by the sensor topology and dynamic components determined by real-time monitoring situations and unexpected anomalies. Inspired by [16], we design a feature block divided into two parts, the disentangled graph convolution layer and the local attention layer, to model the multiscale causal relationships between sensors dynamically over time. We use the disentangled method in the disentangled graph convolution layer to model complex sensor relationships by performing multiscale aggregation on the sensor relationship graph. The disentangled method can eliminate redundant dependencies in the sensor relationship graph and thus decompose different neighborhoods under multiscale aggregation node importance. The local attention layer models time-varying depth dependencies and dynamically captures directional dependencies between sensors using real-time sensor monitoring values, sensor location embedding, and temporal information. Based on considering the correlation of different scales of different time steps, the temporal block based on transformer [17] learns the hidden long-term time-varying relationship from the long-term and short-term dependence through the self-attention mechanism, which promotes the prediction ability of time series and improve the anomaly detection level of the model. Experiments on three real datasets show that the proposed model significantly outperforms recent state-of-the-art models.

The main contributions of this paper are summarized as follows:

1. We design a novel method that eliminates redundant dependencies in the sensor relationship graph by combining a disentangled method with a graph convolution method, enabling a powerful multiscale aggregator to capture fixed correlations between sensors on a time series effectively. At the same time, the method also models highly dynamic inter-sensor correlations. It captures hidden feature patterns of multivariate time series, alleviating the deficiency of graph convolution models in modeling correlations between sensors and accurately describing changes in temporal dynamics in multivariate time series.
2. We propose a method for capturing long-term temporal dependencies that learns hidden long-term temporal dependencies by considering multiscale correlations at different time steps and can be easily extended to long sequences by processing remote dependencies in parallel.
3. Combining inter-sensor dependencies with long-term time dependencies yields a robust anomaly detection model ($D^{3}TN$) with multiscale receptive fields across sensor and time dimensions. The designed disentangled method further enhances the model’s performance while the model has good interpretability.

The remainder of the paper is organized as follows. We briefly review the most relevant work in Section 2. We describe the anomaly detection problem for multivariate time series in Section 3 and describe the proposed solution in detail. Extensive comparative experiments on benchmark datasets are conducted in Section 4 to evaluate our model. Finally, we conclude the paper and discuss further work in Section 5.

## 2. Related Work

We provide a brief overview of existing time series anomaly detection methods and disentangled representational learning and transformer applications.

### 2.1. Anomaly Detection in Time Series

The initial research focused on univariate time series anomaly detection, with the data being treated as anomalies when they deviated from the overall distribution at a specific point. Univariate time series detection methods can detect anomalies in multivariate time series by monitoring each sensor individually [5,6,18]. Univariate time series detection methods can detect anomalies in multivariate time series by monitoring each sensor individually. However, they often lead to poor performance due to the inherent complex linkages between sensors [19]. As transportation, healthcare, and finance become fully digitized, the number of networked sensors and actuators in real-world systems grows [20], and multivariate time series anomaly detection is gradually replacing univariate time series anomaly detection.

The rise and application of neural networks have successfully driven research in pattern recognition and data mining [21,22]. Moreover, by considering multiple sensor features simultaneously, deep learning methods have become a popular approach [23]. Munir et al. [11] first used an unsupervised deep learning method to detect time series anomalies, using a deep convolutional neural network to predict the next timestamp in a defined range and then classify the corresponding timestamp as normal or abnormal based on the predicted value. Su et al. [9] presented a reconstruction-based strategy whose main idea is to develop a robust representation of multivariate time series and then reconstruct the input data. The reconstruction probabilities are used to detect anomalies and anomaly explanations. An LSTM-based variational self-encoder for multimodal anomaly detection is proposed by Park et al. [10]. Encoding involves projecting the observations and time dependency of each time step into the potential space, and decoding involves estimating the predicted distribution based on the representation of the potential space. Audibert et al. [24] proposed a multivariate time series unsupervised approach based on back-trained autoencoders, which has greater generalization capabilities, by combining the advantages of autoencoders and adversarial training.

Many problems, in reality, can be abstracted into graph-structured non-Euclidean data, such as multimedia networks, chemical structures, and transportation networks [25]. Multivariate time series are also considered graph-structured data, and sensors as nodes in the graph have attracted much attention [12]. Deng et al. [13] applies the same concept to discover multivariate time series anomalies by integrating structure learning methods with graph neural networks and applying attention weights to give interpretability of found anomalies. Zhao et al. [14] used graph attention models to learn complex dependencies of multivariate time series based on time and feature dimensions, thus obtaining a better time series representation.

### 2.2. Disentangled Representation Learning

Disentangled representation learning is a well-studied topic that tries to learn the representation of various independent components hidden behind the data. It has been applied to computer vision [26], recommendation [27], natural language processing [28], and other domains. Hamaguchi et al. [29], for example, used the disentanglement technique to detect rare events and proposed a new method for learning disentangled representations from low-cost negative samples in a pair of observations disentangles as variant and invariant factors, respectively, representing mixed information related to trivial events and image content invariant to trivial events. Inspired by the corresponding, researchers have introduced it into the field of data mining. Wang et al. [30] found useful information formed by various hidden factors from multimodal data, i.e., learning from different modalities with complementary and common information in the disentangled representation. Yamada et al. [31] proposed an unsupervised model to learn disentangled representations from sequential data by using the information bottleneck principle to disentangle factors in sequential data into dynamic and static data.

### 2.3. Transformer

In recent years, the transformer has become not only a mainstream model in natural language processing but is also widely used in various fields such as computer vision, recommendation, and time series prediction to achieve optimal performance on multiple tasks. For the picture restoration challenge, Liang et al. [32] adopted the transformer, which not only outperformed convolutional neural network-based image restoration approaches in terms of performance but also led to a significant reduction in model parameters. Chen et al. [33] took into account the sequence nature of user behavior sequences and employed the transformer to record information about prior user behavior sequences, as seen in Taobao’s recommendation scenario. Lim et al. [34] developed a new transformer-based architecture that employs a recursive layer for local processing. The overall architectural design further includes components for selecting relevant features, a gating unit for suppressing redundant elements, and an explainable self-attentive layer for long-range dependencies. Liu et al. [35] proposed a multi-resolution pyramid attention mechanism for long-range dependency modeling and time series prediction, in which an inter-scale tree structure summarizes features at different resolutions and intra-scale adjacent connections have temporal dependencies at different scales, reducing the maximum length of a signal traversal path to O(1) while maintaining linear time and space complexity.

The method proposed in this paper differs significantly from the above methods as follows.

First, our model is unlike the graph neural network-based models [12,13,14]. While the latter uses local information from the learned adjacency matrix to learn inter-sensor dependencies and ignores the complex hidden correlations between sensors, we model the multiscale dynamic dependencies between sensors through a combination of fixed and dynamic features. Second, our model also differs from models that use recurrent neural networks to capture temporal dependencies [7,8,10,11]. Inspired by transformer [32,34], we learn hidden time-varying relationships from long- and short-term dependencies by projecting temporal features into a high-dimensional space through a self-attention mechanism that captures correlations at different scales for different time steps.

## 3. The Proposed $D^{3}\mathrm{TN}$ Model

### 3.1. Problem Formulation

**Figure 1:**
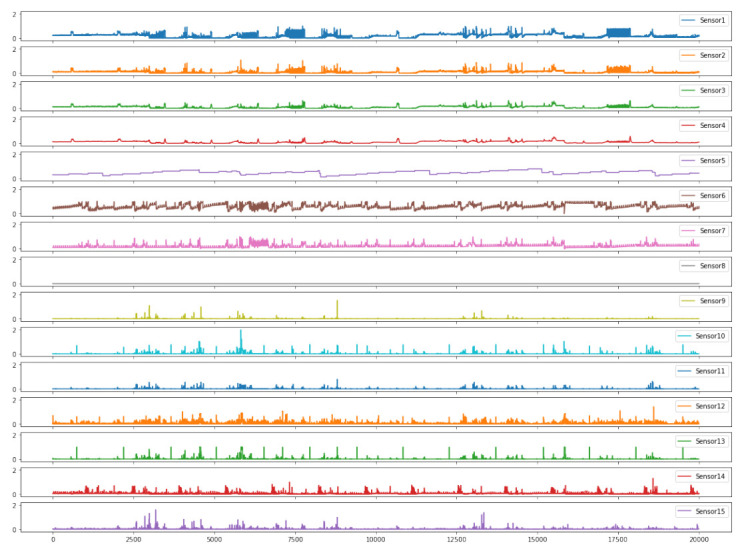
An example of a multivariate time series input. Each column represents the value of the multivariate time series at timestamps, while each row represents a sensor detection value.

In this paper, the input sequence consists of sensor data from sensors over time. The input sequence $X=\left\{x_{1},x_{2},\cdots ,x_{n}\right\}\in R^{n\times m}$, where *n* is the length of the time series, *m* is the number of sensors that generate the multivariate time series, and R represents the set of real numbers. Figure 1 depicts a sample of multivariate time series data consisting of 15 sensors’ monitoring values, where the time series length is 20,000. At arbitrary one-time stamp *t*, an *m*-dimensional vector $x_{t}\in R^{m}$ is formed, representing the values of the m sensors. Our purpose is to detect anomalies by generating an output vector $\left\{y_{1},y_{2},\cdots ,y_{n}\right\}\in R^{n}$, where $y_{t}\in \left\{0,1\right\}$ indicates whether the timestamp *t* is anomalous.

Due to the relatively enormous amount of time series data, we only take the data $\left\{x_{t-w},x_{t-w+1},\cdots ,x_{t-1}\right\}\in R^{w\times m}$ of the first *w* timestamps at moment *t* to predict the value ${\hat{x}}_{t}$ in each iteration, and consider the error between the true value $x_{t}$ and the predicted value ${\hat{x}}_{t}$ as the anomaly score. The higher the anomaly score at time *t*, the more likely an abnormality will occur. It is decided that an anomaly has occurred at time *t* if the anomaly score exceeds the threshold value automatically defined by the dynamic deviation scoring module.

### 3.2. Overall Architecture

**Figure 2:**
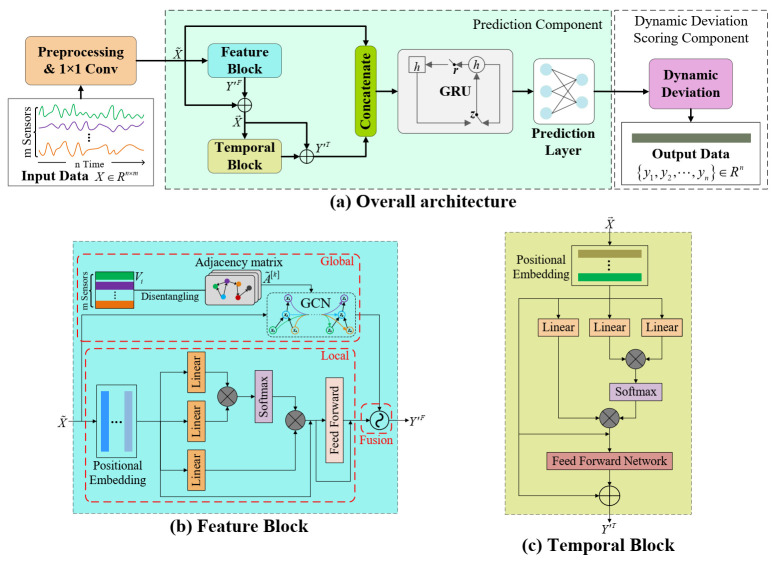
The proposed model architecture, which consists of a prediction module and a dynamic deviation module. The prediction module uses a feature block, temporal block, and GRU to jointly model the inter-sensor dependencies and time dependencies. Skip connections are used to combine all levels of features.

To address the following flaws in previous work: (1) insufficient consideration of complex and variable inter-sensor dependencies in multivariate time series; (2) lack of modeling of long-term temporal dependencies in multivariate time series. In this paper, we propose the $D^{3}TN$ model with the overall architecture shown in Figure 2a, which consists of a prediction component and a dynamic deviation scoring component. In particular, the prediction component uses dynamic multiscale inter-sensor dependence and long-term time dependence for accurate time series prediction. The dynamic deviation scoring component determines whether anomalies occur at a given moment by measuring the difference between predicted and true values. The prediction component integrates three parts: the feature block, temporal block, and GRU [36]. Meanwhile, the correlations between the sensor and temporal dimensions are extracted dynamically and uniformly, and the dependency contexts are accurately predicted. The feature block captures hidden fixed and dynamic inter-sensor dependencies patterns, while the temporal block is designed to learn long-term temporal dependencies efficiently. We concatenate the processed raw data and the output of the feature block and temporal block and then feed them into a recurrent neural network GRU with complex recurrent hidden units for capturing sequential patterns in the fused time series data. Subsequently, the prediction layer stacked by two fully connected layers is used to aggregate the outputs of the GRU layers for time series prediction. Residual connections are added to avoid gradient disappearance during stable training. Finally, the predicted and observed values are fed to the dynamic deviation scoring module, and anomalies are considered to have occurred at that moment if the anomaly score exceeds an adaptive deviation threshold.

### 3.3. Feature Block

In this paper, we treat each sensor as a single node in the graph and learn the dependencies between the nodes. As shown in Figure 2b, the feature block consists of disentangled global graph convolution layer, local attention layer, and feature fusion layer. The dependencies between sensors are complex and variable. Inter-sensor dependencies over a period of time can be disentangled into global components determined by sensor topology and local components determined by real-time monitoring conditions and unexpected anomalies. We develop a deconvoluted global graph convolution layer and a local attention layer to explore the inter-sensor dependencies’ global and local components. Finally, both are sent to the feature fusion layer to capture the hidden dependency patterns of sensors in the time series.

#### 3.3.1. Disentangled Global Graph Convolution Layer

Inherent and nonlinear directional strong connections exist between sensors [37]. Considering the existence of different characteristics among different sensors, we introduce an embedding vector $V\in R^{m\times d}$ for the *m* sensors in the multivariate time series, and the embedding vector *V* is randomly initialized and trained along with the rest of the model, where $V_{i}\in R^{d}$ denotes the embedding vector for the features of sensor *i*.

These embeddings are utilized to calculate the degree of similarity in sensor behavior, as there should be a greater tendency to trend between sensors with similar embedding vectors [38]. Since the multivariate time series has no explicit a priori information about the graph’s structure, all sensors except sensor *i* are candidate sensors on which it can rely. The similarity between sensor *i* and the candidate sensors *j* is defined below:

(1) $$e_{j,i}=\frac{{V_{i}}^{⊤}V_{j}}{∥V_{i}∥\cdot ∥V_{j}∥}\;\mathrm{for}\;j\in \left\{1,2,\cdots ,m\right\}\backslash \left\{i\right\}$$

Although the GCN can perform feature extraction well, it suffers from the problem of over-smoothing on node features, especially in scenarios with plentiful sensors. In addition, the GCN does not clearly and effectively utilize multiscale information beyond superimposing multiple layers. In contrast, an effective multiscale scheme enables the model to remain invariant as the scale changes and capture more intrinsic patterns [39]. The paper [40] uses *k*-order polynomials of adjacency matrices to feature aggregate multiscale structural information and learn rich representations by establishing relationships between distant and near neighbors in this way, and similarly in [26,27,29].

We attempted to apply the method proposed in the paper [40] directly to multivariate time series anomaly detection, but the problem of weight bias between sensors arises when providing a multiscale aggregation operation for the adjacency matrix. It is worth noting that prediction-based anomaly detection models predict normal values of metrics based on historical data and detect anomalies based on predicted deviations and predefined thresholds [7]. When anomalies occur in the system, strong connections are formed between the anomaly sensors, and the edge weights grow exponentially. This situation will cause the model to also learn the pattern of data distribution under anomalies, making our model more biased towards its true value in predicting anomalous data, thus reducing the gap between the predicted and true values.

To effectively alleviate the above problem, inspired by action recognition [16], we propose an inter-sensor dependency aggregation method for disentangling multiple scales. To begin with, we define the adjacency matrix ${\tilde{A}}^{\left[k\right]}$ based on the similarity scale *k* as:

(2) $${\left[{\tilde{A}}^{\left[k\right]}\right]}_{i,j}=\left\{\begin{array}{cc} 1 & \mathrm{if}\;\Delta \left[k\right]\leq e_{i,j}\leq \Delta \left[k+1\right],\\ 1 & \mathrm{if}\;i=j,\\ 0 & \mathrm{otherwise}, \end{array}\right.$$

where $e_{i,j}$ gives the similarity of behavior between sensor *i* and sensor *j*, we set the scale to $\Delta \in \left\{0.2,0.4,0.6,0.8,1\right\}$. ${\tilde{A}}^{\left[k\right]}$ can be represented as a set of unweighted subgraphs. We improve on the GCN used in the paper [40] with the original layer-by-layer update rule as follows:

(3) $${\tilde{X}}^{\left(l+1\right)}=\sigma \left(\sum_{k=1}^{K}{\hat{A}}^{k}{\tilde{X}}^{\left(l\right)}W_{\left(k\right)}^{\left(l\right)}\right),$$

where *K* denotes the number of scales to be aggregated, ${\hat{A}}^{k}={\tilde{D}}^{k-\frac{1}{2}}{\tilde{A}}^{k}{\tilde{D}}^{k-\frac{1}{2}}$ is the normalized form of ${\tilde{A}}^{k}$, ${\tilde{A}}^{k}=A^{k}+I$ adds a self-loop to the adjacency matrix, ${\tilde{D}}^{k}$ is the degree matrix of ${\tilde{A}}^{k}$, $\tilde{X}$ is the input data of the feature block, *W* denotes the weight matrix of the layer learnable, *l* is the index of the layer, and $\sigma \left(\cdot \right)$ is the activation function.

Replacing ${\tilde{D}}^{k}$ in Equation (3) with the disentangled adjacency matrix ${\tilde{A}}^{\left[k\right]}$, we obtain the GCN update rule in this paper:

(4) $${\tilde{X}}^{\left(l+1\right)}=\sigma \left(\sum_{k=1}^{K}{\tilde{D}}^{\left[k\right]-\frac{1}{2}}{\tilde{A}}^{\left[k\right]}{\tilde{D}}^{\left[k\right]-\frac{1}{2}}{\tilde{X}}^{\left(l\right)}W_{\left(k\right)}^{\left(l\right)}\right),$$

where ${\tilde{D}}^{\left[k\right]}$ is the degree matrix of ${\tilde{A}}^{\left[k\right]}$.

Generally, if the associations between sensors are considered fully connected, it is obvious that a large amount of irrelevant information is introduced, thus leading to over-smoothing problems. Adaptively aggregating the information related to the scale range can effectively alleviate the over-smoothing problem. According to the disentanglement method proposed in Equation (4), dependencies are formed if the similarity between sensors is between $\Delta \left[k\right]\leq e_{i,j}\leq \Delta \left[k+1\right]$. By adjusting the range of the similarity scale, the sensors can be associated with different sensors, thus alleviating the weight bias problem.

#### 3.3.2. Local Attention Layer

The GCN-based methods model only the fixed features among sensors and ignore the hidden dynamic dependencies that the time evolution. To exploit the local inter-sensor dependencies that change dynamically over time, we implement training and modeling by learning to represent the input features of each node in a hidden high-dimensional subspace. The input features are projected into the high-dimensional latent subspace through a self-attention mechanism to efficiently model the local dynamic dependencies between sensors based on the changing high-dimensional signals.

The transformer uses self-attention instead of a recurrent network, which results in the inability to obtain information about the relative positions of the observations. Therefore, we represent the location information between nodes in an embedding, and ${\hat{D}}^{F}\in R^{m\times d}$ represents the sensor location embedding matrix, which is randomly initialized and then trained with the model. ${\hat{D}}^{F}$ tiled along the spatial axis to generate $D^{F}\in R^{w\times m\times d}$, we connect the input data $\tilde{X}$ with the sensor position embedding matrix $D^{F}$ to obtain the d-dimensional embedding features $X^{\prime }{}^{F}\in R^{w\times m\times d}$. For simplicity, we consider $X^{F}\in R^{m\times d}$ of $X^{\prime }{}^{F}$ to be described for any timestamp.

Considering that the inter-sensor dependencies, in reality, are complex and changeable, and the occurrence of anomalies is unpredictable, only capturing the fixed dependencies cannot adequately represent the hidden dependencies between sensors. Therefore, we consider multiple linear maps to model the time-varying orientation dependencies of the sensor affected by various factors. We first project the embedding features $X^{F}\in R^{m\times d}$ of each timestamp into three high-dimensional potential subspaces via a feedforward neural network.

(5) $$\begin{array}{c} Q^{F}=X^{F}W_{q}^{F} \end{array}$$

(6) $$\begin{array}{c} K^{F}=X^{F}W_{k}^{F} \end{array}$$

(7) $$\begin{array}{c} V^{F}=X^{F}W_{v}^{F} \end{array},$$

where $W_{q}^{F}\in R^{d\times d^{F}}$, $W_{k}^{F}\in R^{d\times d^{F}}$, $W_{v}^{F}\in R^{d\times d}$ are the weight matrices of the feature query subspace $Q^{F}\in R^{m\times d^{F}}$, feature key subspace $K^{F}\in R^{m\times d^{F}}$, and feature value subspace $V^{F}\in R^{m\times d}$, respectively.

We use dot product self-attention to learn local dependencies $M^{F}$ among multivariate time series sensors, which are further aggregated with $V^{F}$ to obtain the learned node features $Z^{F}$.

(8) $$\begin{array}{c} M^{F}=softmax\left(\frac{Q^{F}{\left(K^{F}\right)}^{⊤}}{\sqrt{d^{F}}}\right) \end{array}$$

(9) $$\begin{array}{c} Z^{F}=M^{F}V^{F} \end{array}$$

Different patterns of inter-sensor dependencies influenced by various factors can be learned here utilizing multi-headed attention, namely the ability to capture different local time-varying dependencies from different high-dimensional potential subspaces. To further improve the predictive power of the model for time series, we input the node features $Z^{\prime }{}^{F}=Z^{F}+X^{F}$ with the addition of residual connections into a shared two-layer feedforward neural network to explore the interactions between the node features.

(10) $$U^{F}=Relu\left(Z^{\prime }{}^{F}W_{0}^{F}\right)W_{1}^{F},$$

where $W_{0}^{F}$, $W_{1}^{F}$ is the weight matrix of the feedforward neural network. $U^{F}$ and $Z^{\prime }{}^{F}$ are combined by ${\tilde{Y}}^{F}=U^{F}+Z^{\prime }{}^{F}$ and feature fusion is performed with a gate mechanism.

#### 3.3.3. Feature Fusion Layer

The gate mechanism is developed to fuse the inter-sensor dependencies learned from the disentangled global graph convolution layer and the local attention layer. We multiply the output $X^{G}$ of the disentangled global graph convolution layer. The output ${\tilde{Y}}^{F}$ of the local attention layer by the weight matrix $W_{G}$, $W_{F}$, respectively, which is transformed by the sigmoid activation function and used as the fusion gate *g*.

(11) $$\begin{array}{c} g=sigmod\left(W_{G}X^{G}+W_{F}{\tilde{Y}}^{F}\right) \end{array}$$

(12) $$\begin{array}{c} Y^{F}=gX^{G}+\left(1-g\right){\tilde{Y}}^{F} \end{array}$$

The output of a single timestamp $Y_{F}$ is obtained by gate *g* weighting $X_{G}$ and ${\tilde{Y}}^{F}$, the output of feature block $Y^{\prime }{}^{F}\in R^{w\times m\times d}$ is obtained by connecting the outputs of *w* timestamps and fed into the subsequent temporal block to extract long-term temporal dependencies.

### 3.4. Temporal Block

Feature blocks capture potential causal relationships between multiple sensors from the sensor dimension of multivariate time series, and this section emphasizes the dependencies modeled along the time dimension of multivariate time series.

Figure 2c shows the temporal block proposed in this paper for efficiently capturing long-range temporal dependencies, where we first randomly initialize the time-position embedding matrix ${\hat{D}}^{T}\in R^{w\times d}$ and then flatten it along the time axis to generate $D^{T}\in R^{w\times m\times d}$. Similar to the feature block, $X^{\prime }{}^{T}\in R^{w\times m\times d}$ is obtained from the concatenation of the input features $\vec{X}=\tilde{X}+Y^{\prime }{}^{F}$ and the temporal embedding $D^{T}$. We still model the temporal dependencies of nodes by parallelization, and this section considers the two-dimensional tensor of any timestamp $X^{T}\in R^{w\times d}$ for description.

A self-attention mechanism is used to model the remote dependence of multivariate time series. Analogous to the feature block, we define three high-dimensional subspaces to capture dynamic temporal correlations, namely, the temporal query subspace $Q^{T}\in R^{w\times d^{T}}$, the temporal key subspace $K^{T}\in R^{w\times d^{T}}$, and the temporal value subspace $V^{T}\in R^{w\times d}$.

(13) $$\left[\begin{array}{c} Q^{T}\\ K^{T}\\ V^{T} \end{array}\right]=X^{T}\left[\begin{array}{c} W_{q}^{T}\\ W_{k}^{T}\\ W_{v}^{T} \end{array}\right],$$

where $W_{q}^{T}\in R^{d\times d^{T}}$, $W_{k}^{T}\in R^{d\times d^{T}}$, and $W_{v}^{T}\in R^{d\times d}$ are the learned weight matrices. In addition, we introduce the scaled dot product function to learn the time dependencies within the historical time.

(14) MT=softmaxQTKT⊤QTKT⊤dTdT

Further, we aggregate the time-valued subspace $V^{T}$ with the weights $M^{T}$ to obtain the temporal features $Z^{T}=M^{T}V^{T}$. To explore the potential interactions within $Z^{T}$, we fed it into a two-layer neural network to learn the hidden temporal dependencies. It is worth mentioning that we introduce residual connections $Z^{\prime }{}^{T}=Z^{T}+X^{T}$ in order to perform stable training.

(15) $$U^{T}=Relu\left(Z^{\prime }{}^{T}W_{0}^{T}\right)W_{1}^{T}$$

The output of each sensor node is $Y^{T}=Z^{\prime }{}^{T}+U^{T}$, and the output of temporal block is $Y^{\prime }{}^{T}\in R^{w\times m\times d}$ obtained by connecting the outputs of *m* sensors.

In the temporal block, the current timestamp is associated with any timestamp within the sliding window, which can effectively capture temporal dependencies. Additionally, the temporal block can easily learn remote dependencies in long sequences by changing the window size without sacrificing much computational efficiency.

The inter-sensor and temporal dependencies of the multivariate time series are already included in $Y^{\prime }{}^{T}$ at this point. Similar to the paper [14], we concatenate the captured inter-sensor dependencies, temporal dependencies, and raw data to obtain $X^{C}$, and feed it into the GRU for capturing sequential patterns in the time series.

### 3.5. Prediction and Model Training

The structure of GRU inputs and outputs is akin to that of a typical recurrent neural network, where the internal ideas are familiar to the LSTM [41]. However, the GRU has no additional storage units to hold information and fewer parameters compared to LSTM. We use the GRU to capture the sequential patterns in the fused data $X^{C}$, and the hidden state of its recursive unit at timestamp *t* is computed as:

(16) $$\begin{array}{c} z_{t}=sigmod\left(W_{z}X_{t}^{C}+U_{z}h_{t-1}\right) \end{array}$$

(17) $$\begin{array}{c} r_{t}=sigmod\left(W_{r}X_{t}^{C}+U_{r}h_{t-1}\right) \end{array}$$

(18) $$\begin{array}{c} {\tilde{h}}_{t}=\tanh\left(WX_{t}^{C}+U\left(r_{t}⊙h_{t-1}\right)\right) \end{array}$$

(19) $$\begin{array}{c} h_{t}=\left(1-z_{t}\right)h_{t-1}+z_{t}{\tilde{h}}_{t} \end{array}$$

Here, $X_{t}^{C}$ is the input of the GRU at moment *t*, ⊙ is an element-wise multiplication. The update gate $z_{t}$ determines the extent to which the cell updates its activation, and the reset gate $r_{t}$ is calculated similarly to the update gate. In addition, the candidate activation ${\tilde{h}}_{t}$ shows the same function as the recursive unit. The activation state $h_{t}$ of the GRU at moment *t* represents a linear interpolation of the current candidate activation ${\tilde{h}}_{t}$ and the previous activation state $h_{t-1}$.

The prediction layer consists of two fully connected layers stacked on top of each other, and predictions are made based on the last layer of the GRU’s output. The prediction $\hat{X}=\left\{{\hat{x}}_{w},{\hat{x}}_{w+1},\cdots ,{\hat{x}}_{n}\right\}\in R^{(n-w)\times m}$ of the model for multivariate time series is obtained by connecting the prediction outputs of all windows.

(20) $$\hat{X}=\left(X^{GRU}W_{0}^{Pre}+b_{0}\right)W_{1}^{Pre}+b_{1}$$

To learn the parameters of the model, we use the root mean square error (RMSE) between the predicted output ${\hat{x}}_{t}$ and the real data $x_{t}$ at moment *t* as the loss function.

(21) $$Loss=\sqrt{\sum_{i=1}^{m}{\left({\hat{x}}_{t,i}-x_{t,i}\right)}^{2}},$$

where ${\hat{x}}_{t,i}$ denotes the predicted value of the *i*-th sensor at moment *t* and $x_{t,i}$ is the true value of sensor *i* at moment *t*. We advance the parameters of the model by stochastic gradient descent and backpropagation on the basis of Equation (21) and update the parameters using the Adam optimizer.

### 3.6. Dynamic Deviation Scoring

We adopt the squared deviation between the predicted value ${\hat{x}}_{t}$ and the actual value $x_{t}$ as the anomaly score, which indicates the degree of deviation of the predicted value of the model from the true value. The higher the anomaly score is, the higher the possibility of anomaly.

(22) $$l_{t}=\sum_{i=1}^{m}{\left({\hat{x}}_{t,i}-x_{t,i}\right)}^{2}$$

A univariate time series $\left\{l_{1},l_{2},\cdots ,l_{w}\right\}$ consisting of anomaly scores are obtained by computing anomaly scores for *w* timestamps within a window. Siffer et al. [42] proposed SPOT, an automatic threshold selection method, which is based on fitting the tail distribution by generalized Pareto (GPD) with the following equation:

(23) $${\bar{F}}_{\tau }\left(l\right)=P\left(L-\tau >l|L>\tau \right)∼{\left(1+\frac{\beta l}{\alpha }\right)}^{-\frac{1}{\beta }},$$

where τ is the initial threshold, α and β are the shape and scale parameters of the GPD, respectively, *L* is any value of $\left\{l_{1},l_{2},\cdots ,l_{w}\right\}$. L−τ follows the generalized Pareto distribution with parameters α and β, indicating the fraction beyond the threshold τ for which a quantile is empirically set.

We adopt the scheme proposed in the literature [43] to speed up the computational efficiency by using the method of moments (MoM) as a parameter estimation method. MoM uses the mean and variance of the sample to estimate the overall and then derives the unknown parameters of the distribution. The mean $E\left(Y\right)=\frac{\alpha }{1-\beta }$ and variance $var=\frac{\alpha^{2}}{{\left(1-\beta \right)}^{2}\left(1-2\beta \right)}$ of GPD can be substituted by $\mu =\sum_{i=1}^{N}\frac{Y_{i}}{N_{t}}$, $S^{2}=\sum_{i=1}^{N}\frac{{\left(Y_{i}-\mu \right)}^{2}}{N_{t}-1}$, correspondingly. Here $Y_{i}$ is the excesses of peaks, $N_{t}$ denotes the number of peaks, i.e., the number of $Y_{i}$ s.t. $Y_{i}>\tau$. The estimates for α and β are calculated by the following equation.

(24) $$\begin{array}{c} \hat{\alpha }=\frac{\mu }{2}\left(1+\frac{\mu^{2}}{S^{2}}\right) \end{array}$$

(25) $$\begin{array}{c} \hat{\beta }=\frac{1}{2}\left(1-\frac{\mu^{2}}{S^{2}}\right) \end{array}$$

The final threshold $\tau_{final}$ is calculated by:

(26) $$\tau_{final}=\tau +\frac{\hat{\alpha }}{\hat{\beta }}\left({\left(\frac{qn}{N}\right)}^{-\hat{\beta }}-1\right),$$

where *q* is the risk factor applied to determine the anomaly.

The final threshold is selected automatically by the POT-MoM, and only two parameters (quantile and risk factor) need to be adjusted empirically. We calculate the most appropriate threshold adaptively using Algorithm 1.
**Algorithm 1** POT-MoM**Input:** input data $\left\{l_{1},l_{2},\cdots ,l_{w}\right\}$, risk *q***Output:** initial threshold τ, final threshold $\tau_{final}$  1:$\tau \leftarrow SetInitialThreshold(\left\{l_{1},l_{2},\cdots ,l_{w}\right\})$  2:$Y_{t}\leftarrow \left\{L_{i}-\tau |L_{i}>\tau \right\}$  3:$\hat{\alpha },\hat{\beta }\leftarrow MoM\left(Y_{t}\right)$  4:$\tau_{final}\leftarrow CalcFinalThredhold\left(q,\hat{\alpha },\hat{\beta },n,N_{t},t\right)$

## 4. Performance Analysis

In this section, we validate the model’s performance using three real anomaly detection datasets to evaluate the proposed model against state-of-the-art methods. We first present the experimental setup and then perform numerous experiments to demonstrate the superiority of our model.

### 4.1. Experiment Setup

We follow the anomaly detection convention of unsupervised learning and assume that the training data consists of normal data only. Considering that the magnitudes of different sensors may be inconsistent and the differences between values vary widely, all data sets are first subjected to min-max normalization to boost the system’s robustness. To alleviate the phenomenon that prediction-based models are susceptible to anomalies in the data, we employ the lightweight unsupervised algorithm SR [44] for data cleaning on the training set only.

#### 4.1.1. Datasets

As shown in Table 1, MSL and SMAP are spacecraft datasets collected and published by NASA [45], each of which has a training subset and a test subset, and both test subsets have labeled files in which all anomalies are marked. SMD [9] is an application server dataset and is the largest public dataset available for evaluating multivariate time series anomaly detection. It contains 28 different servers, each with 38 features representing different metrics of the server (i.e., CPU load, network usage), where the former half of the data is used for training and the other half is used as a test set.

#### 4.1.2. Evaluation Metrics

We adopt the mainstream anomaly detection evaluation metrics precision (P), recall (R), and F1-score (F1) to evaluate the performance of each model, and the higher the value of the three metrics, the stronger the performance of the model. The calculation method is as follows:

(27) $$\begin{array}{c} P=\mathrm{TP}/\left(\mathrm{TP}+\mathrm{FP}\right) \end{array}$$

(28) $$\begin{array}{c} R=\mathrm{TP}/\left(\mathrm{TP}+\mathrm{FN}\right) \end{array}$$

(29) $$\begin{array}{c} F1=\frac{2\times P\times R}{P+R} \end{array},$$

where TP and FP denote true positives and false positives, respectively, and FN refers to false negatives.

In practice, an abnormality in one indicator usually leads to abnormalities in other indicators, which will form an abnormal segment over a period of time, and any timestamp of an abnormal segment triggering an abnormal alarm is acceptable. In this paper, we use the point adjustment method proposed by Xu et al. [46] to calculate the model’s performance, widely applied for evaluating time series anomaly detection tasks [9,14,24,37,44]. More specifically, if any anomaly in an anomalous segment of the test set is detected, we believe that threshold can detect all the anomalies in that segment. In contrast, the points outside the anomalous segment are not additionally processed.

#### 4.1.3. Experimental Scheme

We evaluate the performance of the designed model in the following aspects through extensive experiments.

1. Comparison with existing state-of-the-art methods. We validate the effectiveness of the proposed model by comparing it with six state-of-the-art anomaly detection models.
2. Visualize model training results. We select some results from single server monitoring data on the SMD dataset to highlight the model’s excellent performance and provide support for model comparison analysis.
3. Ablation experiments. In order to verify the validity of the components that make up the model, we design ablation experiments to remove components one by one and keep all experimental environments consistent.
4. Anomaly explanation. The ability of the model to accurately provide valuable insights for anomaly detection is an important metric. These insights can help operators troubleshoot quickly and save problem-solving effort.

#### 4.1.4. Baselines

To verify the effectiveness of the models proposed in this paper more comprehensively, we have chosen the following typical anomaly detection algorithms as a comparison method. These are the most popular detection algorithms, including prediction-based and reconstruction-based models, which have been published in top conferences and journals over the past five years.

1. LSTM-NDT [7]: An unsupervised threshold determination method is proposed for anomaly detection of multivariate time series using LSTM.
2. LSTM-VAE [10]: LSTM replaces the feedforward network in VAE by modeling the underlying distribution of the multidimensional signal and then reconstructing the signal with the desired distribution information, using the negative log-likelihood of the reconstructed observation distribution as the anomaly score.
3. OmniAnomaly [9]: Capturing the normal patterns of multivariate time series by learning a robust representation of them and then reconstructing the input data using the reconstructed probabilities as anomaly scores.
4. MAD-GAN [8]: LSTM-RNN is used as the base model for GAN learning to capture the temporal dependence, the discriminator and generator of GAN are used to detect anomalies, and the discriminative results and reconstruction bias of the test samples are combined to calculate the anomaly score.
5. MTAD-GAT [14]: A prediction-based and reconstruction-based model is jointly optimized using two parallel GAT layers that dynamically learn the relationship between different time series and timestamps.
6. USAD [24]: Building an encoder–decoder architecture using a self-encoder that utilizes an adversarial training strategy to learn how to amplify the reconstruction bias of anomalous inputs is more stable than with the traditional GAN-based approach.

#### 4.1.5. Parameter Settings

We refer to the original literature on the comparison algorithms as well as the experimental results, and the optimal performance is obtained for each comparison algorithm. In our method, we are using sliding windows of size 50 and 80 in MSL, SMAP dataset, and window size 100 in the SMD dataset. The length of the sensor embedding vector is 32 and the hidden dimension of the GRU layer and the prediction layer is 150. We train the models for 50 batches, with batch size set to 64 and the initial learning rate of 0.0002. Since the performance of the model is directly affected by the threshold, we select the appropriate quantile and risk factor for each data set by a grid search to achieve optimal performance.

### 4.2. Performance Comparison

As shown in Table 2, our model has excellent generalization ability and consistently achieves the best F1-scores on all datasets, unmatched by other baselines. Specifically, we achieve 2.9%, 4.3%, and 0.7% improvement over the best state-of-the-art performance on the MSL, SMAP, and SMD datasets, respectively. From Table 2, we can derive the following observations.

1. LSTM-NDT performs the worst on the MSL and SMD datasets, while LSTM-VAE performs the worst on the SMAP dataset. This is reasonable since LSTM-NDT only considers the temporal patterns of univariate time series and ignores the inter-sensor dependencies, which leads to its inability to make accurate predictions when the dataset has numerous sensors.
2. In contrast, OmniAnomaly models inter-sensor dependencies by stochastic methods, and MAD-GAN and USAD use adversarial training to learn inter-sensor dependencies, all achieving better performance. However, they ignore the low-dimensional representation of the temporal dimension and perform poorly in modeling temporal dependencies. All three methods are based on reconstructed models, and they reconstruct the data within the window as well as possible during the training learning process. In reality, the training data contains anomalous data. USAD performs the best among these three methods, and we speculate that USAD compensates for this shortcoming by combining the advantages of both autoencoder and adversarial training to improve the detection of anomalies.
3. Unlike the above, MTAD-GAT integrates prediction-based and reconstruction-based models, using graph attention networks to learn temporal and feature dimensions’ dependencies, respectively. However, it assumes that all sensors in the dataset are interdependent, which not only simplifies the complex partial orientation dependencies between sensors but also introduces a large amount of irrelevant information that increases the complexity of the model and thus reduces the performance of the model.

Our model can capture hidden dependency patterns that other models ignore, ensuring the model’s robustness and generality. In contrast, all of the above models do not utilize multiscale dynamic inter-sensor dependencies and long-term temporal dependencies to improve model performance, which is the most significant reason our model outperforms other models.

### 4.3. Performance Visualization

**Figure 3:**
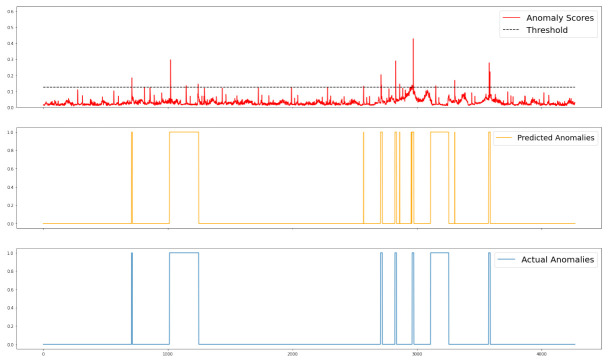
Visualization of entity-level anomaly scores.

To demonstrate the effect of the proposed model more graphically, we chose to visualize a portion of the experimental data. As shown in Figure 3, the red line represents the anomaly score, which indicates the squared error between the expected and actual values. The black dashed line represents the threshold value that the model adaptively builds based on the distribution pattern of the data. The many strange spikes in the anomaly scores represent significant differences between the predicted and actual values. However, these spikes are only identified as anomalies when exceeding the threshold. The orange line depicts the interval of anomalies predicted by the model, while the blue line shows the actual anomaly distribution of the data.

**Figure 4:**
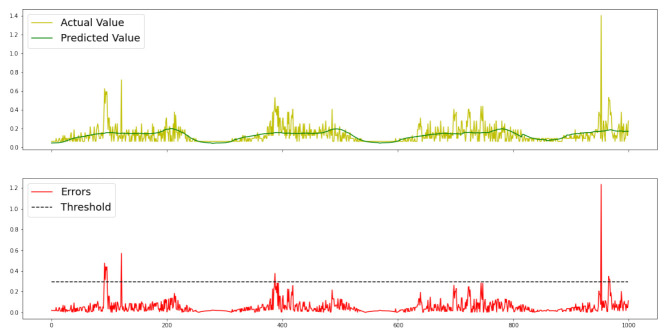
An example of data distribution on the sensor dimension.

Figure 4 shows an example of learning the distribution of sensor data. As shown in Figure 4, the prediction-based model is not good at predicting the raw data but rather the normal value at the next timestamp if possible. This allows us to widen the gap between the predicted and actual values in the anomaly interval and then use the appropriate threshold to make anomaly determinations.

### 4.4. Ablation Experiments

To verify the effectiveness of each model component, we designed several sets of ablation comparison experiments to demonstrate the necessity of the feature block, temporal block, and GRU components. Only the following changes are made among the models, and all other parameters are kept the same.

1. w/o Feature: Remove the feature block, the data is pre-processed and fed straight into the temporal block to capture the time dependence of the time series.
2. w/o Temporal: Remove the temporal block, the data is fed directly into the GRU after output from the feature block.
3. w/o GRU: Remove the GRU and send the learned inter-sensor dependencies and temporal dependencies directly to the prediction layer for prediction.

**Figure 5:**
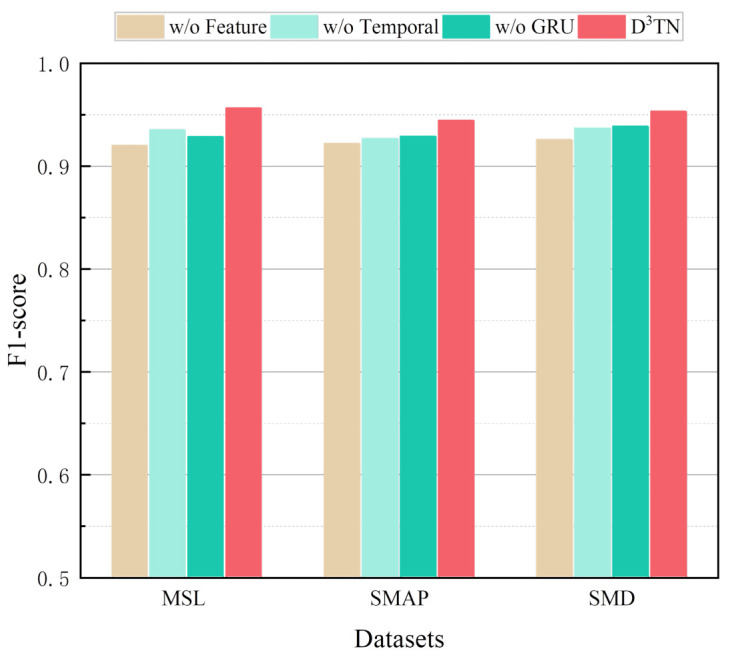
F1-score of our model and the variants.

The performance of the prediction-based anomaly detection model is directly related to the ability of the model to predict multivariate time series. As shown in Figure 5, the w/o Feature model causes the most significant degradation in performance on all three datasets, which is particularly pronounced on the MSL dataset, where the F1-score decreases by 3.64% compared to the entire model, which is good evidence that the multiscale dynamic dependence among sensors can improve the anomaly detection of multivariate time series, since MSL has more sensors and anomaly types than SMAP and SMD. The experimental results of the w/o Temporal model and w/o GRU model verify that the temporal dependencies of time series are also critical for the final performance. In contrast, our proposed model captures long-term temporal dependencies by considering all timestamps within the window through the temporal block and then models the sequential dependencies of time series using the GRU. These experiments illustrate that removing any one component of the model will result in a degradation of performance, whereas by considering all components together, the model will achieve optimal performance.

### 4.5. Anomaly Explanation

In practice, the occurrence of anomalies in multivariate time series is the result of the combined action of multiple sensors. A set of indicators is usually used for anomaly segment interpretation, as the operator can also not find the most anomalous indicator exactly. Hence, the ability to obtain accurate interpretation at the anomaly segment level is vital.

The “InterPretation Score” (IPS) metric was proposed to assess the accuracy of anomaly interpretation at the segment level [21]. Specifically, we compute the contribution value $S_{t}^{i}={\left({\hat{x}}_{t}^{i}-x_{t}^{i}\right)}^{2}\left(1\leq i\leq m\right)$ on a dimension-by-dimension basis for the *m*-dimensional anomalous data. The aggregate number of anomalous segments is N, and for anomalous segment $G_{\Phi }$, the contribution of sensor i to the anomalous segment is defined as $S_{G_{\Phi }}^{i}=\max_{x_{t}\in G_{\Phi }}S_{t}^{i}$. All sensors are placed into a descending list $LS_{G_{\Phi }}$ in order of their contribution values from largest to smallest. The top-ranked sensors in $LS_{G_{\Phi }}$ are more likely to have anomalies and contribute more to the anomaly segment $G_{\Phi }$. The IPS is defined as follows.

(30) $$w_{\Phi }=\frac{\left|G_{\Phi }\right|}{\sum_{\Phi =1}^{N}\left|G_{\Phi }\right|}$$

(31) $$\mathrm{IPS}=\sum_{\Phi =1}^{N}w_{\Phi }\frac{Hit@\left\lfloor P\%\times \left|GT_{\Phi }\right|\right\rfloor }{\left|GT_{\Phi }\right|},$$

where $GT_{\Phi }$ indicates the true cause of the anomaly that caused the data within $G_{\Phi }$, $\left|GT_{\Phi }\right|$ is the number of sensors in $GT_{\Phi }$, $Hit@\left\lfloor P\%\times \left|GT_{\Phi }\right|\right\rfloor$ refers to the number of hits in $LS_{G_{\Phi }}$ for the first sensors in $\left\lfloor P\%\times \left|GT_{\Phi }\right|\right\rfloor$, and $\left|G_{\Phi }\right|$ indicates the total number of anomalies detected in the anomaly segment $G_{\Phi }$. Intuitively, IPS is the weighted sum of the top $\left\lfloor P\%\times \left|GT_{\Phi }\right|\right\rfloor$ of hits evaluated at the segment level [21].

Similar to the papers [9,35,47], we use the SMD dataset for anomaly interpretation experiments because only SMD provides true interpretation labels that cause data anomalies, which indicates that the sensors in the labels are not listed in a specific order and all sensors have equal importance. We compute IPS for each server data separately and take the average of all results as follows. Table 3 illustrates that our model can accurately locate most of the sensors that cause anomalies and that it is feasible to present them as anomaly explanations to the operator in a practical application.

### 4.6. Engineering Applications

**Figure 6:**
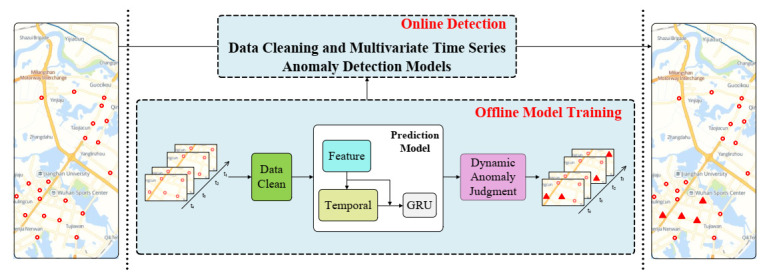
An example of offline model training and online detection process on traffic data.

Traffic data are standard multivariate time series data, and the methods suggested in this work can be integrated into the interconnected systems. As shown in Figure 6, through the offline mode training and online detection, for different systems, we only need to change the data cleaning method to get high-quality input data to train our model directly, set the anomaly threshold offline according to the POT improved by the method of moments. Then the obtained model can reasonably determine whether anomalies occur in observations on a time step. In a prediction-based model, the error between the predicted and actual values is used as the anomaly score. If a single timestamp’s observations follow the time series’s normal pattern, it can be predicted with a low anomaly score. In contrast, the higher the anomaly score, the more significant the departure of the observation from the projected value and the higher the likelihood of it being abnormal.

One of the critical aspects of the problem faced when applied practically to traffic systems is reliable detection and cause diagnosis. For example, the primary goal of traffic anomaly detection is to detect outliers in traffic data. However, the appearance of outliers does not always imply a traffic anomaly event, as external factors such as sensor failure, data noise, and other factors can also cause outliers [48,49]. How to distinguish the difference between outliers caused by these external factors and real urban traffic anomalies remains an unsolved problem. Furthermore, we discovered that it is challenging to utilize it to determine the underlying cause of traffic anomalies since the reason for the anomalies expresses itself in dynamic changes in urban traffic, which is reflected in multiple urban traffic data. In order to find the core cause of anomalous traffic events, it is frequently necessary to infer plausible causal relationships from several traffic data sources spanning time and geography [50]. This challenge has been partially solved by modeling inter-sensor and temporal interdependence together. However, a more lightweight and precise root cause explanation would support the application in more realistic circumstances.

## 5. Conclusions

In this work, we present an unsupervised multivariate time series anomaly detection model. We introduce a novel disentangling multiscale composition method in global graph convolution to collectively model sensor dynamic dependencies at various scales in the local attention layer. At the same time, the model also traps long-term temporal dependencies for improving prediction performance. Finally, anomalies are scored using an improved automatic threshold selection method to capture anomalies. In addition, a generic model-based measure of anomaly interpretation capability proves that our model has good anomaly interpretation capability, which can help operators quickly locate the root cause of anomalies in practice. Future work can consider two points: (1) How to improve the model in practical engineering applications with reliable detection and root cause diagnosis problems and apply it to more complex practical scenarios to improve the model’s utility further. (2) Although the unsupervised learning technique for anomaly identification presupposes that the training data is entirely normal, anomalies exist in the training set and can have negative consequences. As a result, it is critical to ensure the model is trained with as much normal and consistent data as possible for anomaly identification. Pre-training is particularly common in natural language processing and images, and for future work, we consider further pre-training to improve the model’s anomaly detection ability.

## Figures and Tables

**Table 1:** Statistics of the dataset.

Dataset	#Entities	#Features	Train	Test	Anomaly
MSL	55	25	58317	73729	10.72%
SMAP	27	55	135183	427617	13.13%
SMD	28	38	708405	708420	4.16%

**Table 2:** Performance comparison of the 3 datasets using precision (P), recall (R), and F1-score (F1). The top results are highlighted in bold and the secondary results are underlined.

Methods	MSL			SMAP			SMD		
	**P**	**R**	**F1**	**P**	**R**	**F1**	**P**	**R**	**F1**
LSTM-NDT	0.5934	0.5374	0.5640	0.8965	0.8846	0.8905	0.5684	0.6438	0.6037
LSTM-VAE	0.5257	0.9546	0.6780	0.8551	0.6366	0.7298	0.8698	0.7879	0.8268
OmniAnomaly	0.8867	0.9117	0.8989	0.7416	0.9776	0.8434	0.8334	0.9449	0.8857
MAD-GAN	0.8517	0.8991	0.8747	0.8049	0.8214	0.8131	0.9230	0.8694	0.8982
MTAD-GAT	0.8754	0.9440	0.9084	0.8906	0.9123	0.9013	0.9396	0.9283	0.9339
USAD	0.8810	0.9786	0.9272	0.7697	0.9831	0.8634	0.9314	0.9617	0.9463
$D^{3}TN$	0.9454	0.9673	**0.9562**	0.9534	0.9350	**0.9441**	0.9356	0.9709	**0.9529**

**Table 3:** Explanation of exceptions.

	IPS@100%	IPS@150%
SMD	0.7984	0.8923

## Data Availability

This research employed publicly available datasets for its experimental studies.
